# The future of pharmacy is intertwined with digital health innovation

**DOI:** 10.1177/17151635211044474

**Published:** 2021-09-20

**Authors:** Peter Chengming Zhang

**Affiliations:** Peter Chengming Zhang is a candidate in the combined Doctor of Pharmacy/MBA program at the University of Toronto’s Leslie Dan Faculty of Pharmacy and the Rotman School of Management

The integration of new technologies to optimize pharmacy workflow has been transformative. In recent decades, the development of a computerized workflow has allowed pharmacists to automate the process of identifying drug interactions, readily access online therapeutic resources and digitize the storage of patient information.

For the traditional pharmacy workflow, which includes activities such as medication dispensing, providing medication counselling to patients and responding to drug information consults, pharmacy technology solutions have greatly enhanced the capacity for pharmacist productivity. However, while these advancements have increased the efficiency of the traditional pharmacy workflow, newer tasks brought on by expansions of pharmacist clinical services have been relatively untouched by technology.

In recent years, the emphasis within pharmacy has shifted away from product-oriented services and instead toward the provision of clinical care. With the introduction of services such as medication reviews, vaccinations, COVID-19 testing and prescribing for ambulatory conditions, the movement toward a clinical focus in pharmacy is well underway.

However, due to the limitations of time available to pharmacy practitioners on an average workday, there are constraints to introducing new clinical services as a routine aspect of the pharmacist role. Without the concurrent advancement of digital health technologies to support expansions in clinical services, further growth of professional scope may become unsustainable. This places pharmacists in a difficult situation in which time constraints can affect the quality or quantity of services provided. For example, the time necessary to complete documentation after conducting medication reviews in Ontario results in a significant amount of time away from patient care.

With ongoing pressures to perform their usual tasks, practitioners may have no choice but to sacrifice the quality or quantity of medication reviews and other clinical care duties, especially since they are often conducted without an advance appointment. Furthermore, without the intervention of digital products to make the provision of medication reviews more efficient, the feasibility of this clinical service as a routine part of the pharmacist role is uncertain.

Other clinical services, such as the administration of vaccinations, also face similar challenges when integrated within the pharmacist workflow. However, there have been recent changes in this area that have alleviated pressures on the pharmacist workload. In the ongoing COVID-19 pandemic, pharmacy services like COVID-19 testing have been developed as an appointment-based service, and existing procedures such as walk-in flu shots have been changed to scheduled injections. Importantly, these developments demonstrate that pharmacy services can thrive when an appointment-based system is implemented. This shift toward appointment-based services may represent a lasting change in the way pharmacists carry out services such as vaccinations and presents an opportunity for digital health products to create value.

Clinical activities like medication reviews, COVID-19 testing and administering flu shots can be time-consuming. Furthermore, the unpredictable nature of patients entering a pharmacy can often push pharmacists beyond a sustainable workload. Fortunately, these challenges can be overcome with novel digital health products that adequately support changes in pharmacist needs. In one example, Quality Medication Reviews (QMR) Pharmacy Solutions, a digital health company led by pharmacists, seeks to increase the efficiency of the documentation process for medication reviews in Ontario. Its product intends to automate repeated inputs and allows for the completion of the documentation process online. If this concept is successfully implemented, it will allow pharmacists to spend more time with patients and less time on paperwork.

In another example, recognizing the need for scheduled pharmacy services during COVID-19, pharmacist-led companies such as MedEssist and MedMe have developed digital platforms to allow patients to book flu shots or COVID-19 tests online. With the capacity to schedule their services with ease, pharmacists are able to arrange appropriate staffing while avoiding unexpected crowds at the counter and ultimately, keep the pharmacy and its patients safe as the pandemic progresses.

These are just a few of the numerous examples of pharmacist-led digital health solutions. As health technology innovators, pharmacists are uniquely situated not only due to their training as medication experts but also as expert navigators of the health care system. As a result, digital products developed by pharmacists are patient oriented and more user-friendly for clinicians. These attributes are critical in bringing a successful digital health product into the market.

With continuous expansions in the scope of clinical services offered by pharmacists, the integration of digital health within the field of pharmacy will become increasingly necessary. With the incorporation of automation on the horizon, we should embrace digital health as a tool that allows us to do more with our time. We need pharmacists to serve not only as a consultation group for novel health technologies but also as leaders in their innovation.

